# Adoption of Emergency Department–Initiated Buprenorphine for Patients With Opioid Use Disorder: Secondary Analysis of a Cluster Randomized Trial

**DOI:** 10.1001/jamanetworkopen.2023.42786

**Published:** 2023-11-10

**Authors:** Evangeline Gao, Edward R. Melnick, Hyung Paek, Bidisha Nath, R. Andrew Taylor, Andrew J. Loza

**Affiliations:** 1 Department of Emergency Medicine, Yale University School of Medicine, New Haven, Connecticut; 2 Yale School of Public Health, New Haven, Connecticut; 3 Information Technology Services, Yale New Haven Health, Stratford, Connecticut

## Abstract

**Importance:**

Emergency department (ED) initiation of buprenorphine is safe and effective but underutilized in practice. Understanding the factors affecting adoption of this practice could inform more effective interventions.

**Objective:**

To quantify the factors, including social contagion, associated with the adoption of the practice of ED initiation of buprenorphine for patients with opioid use disorder.

**Design, Setting, and Participants:**

This is a secondary analysis of the EMBED (Emergency Department-Initiated Buprenorphine For Opioid Use Disorder) trial, a multicentered, cluster randomized trial of a clinical decision support intervention targeting ED initiation of buprenorphine. The trial occurred from November 2019 to May 2021. The study was conducted at ED clusters across health care systems from the northeast, southeast, and western regions of the US and included attending physicians, resident physicians, and advanced practice practitioners. Data analysis was performed from August 2022 to June 2023.

**Exposures:**

This analysis included both the intervention and nonintervention groups of the EMBED trial. Graph methods were used to construct the network of clinicians who shared in the care of patients for whom buprenorphine was initiated during the trial before initiating the practice themselves, termed *exposure*.

**Main Outcomes and Measures:**

Cox proportional hazard modeling with time-dependent covariates was performed to assess the association of the number of these exposures with self-adoption of the practice of ED initiation of buprenorphine while adjusting for clinician role, health care system, and intervention site status.

**Results:**

A total of 1026 unique clinicians in 18 ED clusters across 5 health care systems were included. Analysis showed associations of the cumulative number of exposures to others initiating buprenorphine with the self-practice of buprenorphine initiation. This increased in a dose-dependent manner (1 exposure: hazard ratio [HR], 1.31; 95% CI, 1.16-1.48; 5 exposures: HR, 2.85; 95% CI, 1.66-4.89; 10 exposures: HR, 3.55; 95% CI, 1.47-8.58). Intervention site status was associated with practice adoption (HR, 1.50; 95% CI, 1.04-2.18). Health care system and clinician role were also associated with practice adoption.

**Conclusions and Relevance:**

In this secondary analysis of a multicenter, cluster randomized trial of a clinical decision support tool for buprenorphine initiation, the number of exposures to ED initiation of buprenorphine and the trial intervention were associated with uptake of ED initiation of buprenorphine. Although systems-level approaches are necessary to increase the rate of buprenorphine initiation, individual clinicians may change practice of those around them.

**Trial Registration:**

ClinicalTrials.gov Identifier: NCT03658642

## Key Points

**Question:**

Does social contagion affect adoption of the practice of emergency department initiation of buprenorphine for opioid use disorder?

**Findings:**

In this secondary analysis of a multicenter, cluster-randomized trial of 5 health care systems and 1026 clinicians, including attending physicians, residents, and advanced practice practitioners, the number of interactions with another clinician initiating buprenorphine in the emergency department had a dose-dependent association with self-adoption of the practice. The primary trial intervention, health care system, and clinician type were also associated with practice adoption.

**Meaning:**

Interaction with other clinicians initiating buprenorphine was associated with increased likelihood of self-adoption, suggesting that there may be a role for social factors in practice uptake.

## Teaser

This secondary analysis of a cluster randomized trial examines the factors, including social contagion, associated with the adoption of the practice of emergency department (ED) initiation of buprenorphine for patients with opioid use disorder.

## Introduction

Opioid use disorder (OUD) is a major public health problem affecting an estimated 2 million Americans.^1,2^ Opioid overdose rates are also increasing, with an estimate of a 4.0% quarterly increase in nonfatal opioid overdoses from 2018 to 2022.^3,4^ With 2.88 million opioid-related emergency department (ED) visits in 2016 and evidence showing continued rate increases, the ED is increasingly viewed as a front-line setting for initiating OUD treatment, such as buprenorphine-naloxone, during a critical period at which patients are at high risk for future overdoses.^3,5,6,7^ Despite this potential role, evidence shows uptake of this practice is low. A survey of 289 physicians across 4 academic ED sites found that only 26% of respondents felt ready to initiate buprenorphine.^8,9^ Barriers cited included difficulty identifying appropriate patients, lack of training in prescribing buprenorphine, uncertainty about evidence, uncertainty about connection to follow-up, and competing priorities in ED care.^8,9^ Outpatient studies examining barriers to adoption have identified similar themes of lack of training and additional barriers related to stigma, role of primary care physicians, and medication cost.^10,11^

Social contagion is the spread of behaviors or attitudes within a group of individuals. It has been implicated in the spread of behaviors and characteristics within populations and studied in the adoption of medical practice.^12,13^ Social factors may impact multiple barriers to buprenorphine adoption by affecting attitudes, setting a social standard, overcoming or reinforcing stigma, or providing direct examples of a specific behavior.^14^

Understanding the factors associated with buprenorphine prescribing is critical to designing interventions to increase initiation, a key step in the OUD continuum of care.^15^ It is estimated that approximately 87% of patients with OUD do not receive medical therapy.^16^ Although surveys identify potential treatment barriers, they are subject to reporting bias and the limitations of self-report. We sought to address this deficiency through a secondary analysis of the Emergency Department-Initiated Buprenorphine for Opioid Use Disorder (EMBED) Trial. This trial examined the effect of a user-centered clinical decision support (CDS) intervention in a pragmatic, parallel, cluster randomized superiority trial in ED clusters across health care systems.^17,18,19,20^ Although the study did not show a significant increase in patients receiving buprenorphine, the number of unique physicians who initiated buprenorphine increased throughout the study, providing a rationale to analyze factors associated with adoption of the practice. The prevalence of buprenorphine use in the ED before the study was low, with sites reporting use rates of 0% to 2%.^21^ We examined the uptake rates of buprenorphine initiation practice for attending physicians, resident physicians, and advanced practice practitioners (APPs) through a time-varying Cox model of buprenorphine initiation in a dynamic network of clinician interactions. By adjusting for key covariates, we also investigated the contribution of health care system, clinician role, and intervention site status on uptake of buprenorphine initiation.

## Methods

### Study Design

This is a post hoc secondary analysis of data collected during the EMBED trial, a cluster randomized trial involving 18 ED clusters across 5 health care systems. Details of the study’s design, including prespecified outcomes, sample size calculations, and randomization procedures, have been published previously.^21,22^ The trial protocol is shown in Supplement 1. EMBED was performed from November 2019 to May 2021, during which on-site buprenorphine administration for durations less than 72 hours (applicable to ED care) did not require an X-waiver, but outpatient prescription of buprenorphine required an X-waiver. It evaluated the effectiveness of a CDS tool to improve physician initiation of buprenorphine in the ED for patients with OUD. The primary trial was approved by the Western Institutional Review Board, and a waiver of informed consent was obtained per the Common Rule (45 CFR 46.116)^21^; the present study was determined to be institutional review board exempt because it was secondary analysis of deidentified data. This study followed the Consolidated Standards of Reporting Trials (CONSORT) reporting guidelines.^23^

### Participants

Eligible patient visits were identified with a validated, electronic health record phenotype that included 2 algorithms. The first was based on *International Statistical Classification of Diseases and Related Health Problems, Tenth Revision* diagnostic codes related to opioid use, and the second was based on chief concerns related to substance use but with no alternative diagnosis related to the use of alcohol or benzodiazepines. For example, a chief concern of withdrawal would be included unless a diagnosis code related to alcohol or benzodiazepine withdrawal was present. These visits were filtered to identify patients aged 18 years or older with probable OUD who were discharged from the ED, not pregnant, and not currently receiving medication for OUD, as reported in the electronic health record.^24^ For patients with multiple visits to the ED during the trial, only the first visit was included in the primary analysis for the EMBED trial, but all visits were included in this secondary analysis. All attending physicians, resident physicians, and APPs (referred to in this article as *clinicians*) on care teams caring for eligible patients were included in the analysis.

### Variables and Constructs for Analysis

The data were used to construct a dynamic network of buprenorphine initiation and clinician interactions to examine individual, environmental, and social factors contributing to buprenorphine initiation. The nodes were defined as attending physicians, residents, and APPs, and the edges were defined as exposures to buprenorphine initiation. Buprenorphine initiation was defined as ordering buprenorphine for ED administration, prescribing buprenorphine for outpatient use, or authorizing either of these orders. During the study period, ED administration or authorization did not require additional certification or training, but outpatient prescription required an X-waiver for all clinicians, which included 8 hours and 24 hours of additional training for physicians and APPs, respectively. An exposure was defined as a clinician who had not yet initiated buprenorphine during the study but was on a care team for a patient who received buprenorphine from another clinician. The status of each node (as adopter vs nonadopter) was a time-dependent binary variable of whether each clinician had initiated buprenorphine during the study period, and conversion time was set to the day of the encounter where this first initiation occurred. This time to clinician status change from nonadopter to adopter was the primary outcome variable. If 2 or more clinicians on a care team initiated buprenorphine for the first time (eg, 1 doing so by ordering and 1 by authorizing), both were considered to convert at that encounter. Additional covariates included in the model were clinician role (APP, attending physician, or resident physician), a categorical variable denoting which health care system the clinician worked within, the intervention status of the site in the EMBED trial (intervention vs control), and the academic site status. Clinicians occasionally worked at multiple sites with differing status (0.5% for health care system, 5% for academic, and 13% for intervention). Site status variables were assigned by the most common site of exposure.

### Statistical Analysis

Data analysis was performed from August 2022 to June 2023. The buprenorphine initiation status of each clinician was modeled as a time-to-event survival process with Cox proportional hazards regression, where the event was the first initiation of buprenorphine by a given physician during the study, referred to as the conversion time. Because buprenorphine prescribing status before the study start date was unknown, this represents a time-to-next event analysis since we could not determine whether a buprenorphine initiation event was a clinician’s first ever initiation of buprenorphine. Social contagion was estimated by using the cumulative number of exposures to buprenorphine initiation as a time-dependent variable to measure of the social force associated with individual likelihood to adopt the practice of prescribing buprenorphine. Two cumulative exposure variables were defined: total cumulative exposures and exposure count in the prior 4 months to account for the fact that exposures before the study start period were not observable. For the 4-month exposure variable, all conversions before 4 months were discarded. To examine nonlinearities in the effect of exposures on hazard of conversion, the cumulative exposure variable was modeled with penalized cubic splines with 2 *df*. Statistical associations between adoption rate and the categorical variables in Table 1 were assessed using the χ^2^ test, with significance set at *P* < .05. Subsequent ordering or prescribing rates after conversion were calculated by dividing the number of observed subsequent prescribing events by the remaining study duration after time of conversion and were normalized to a 90-day interval. Data preprocessing was performed in the Python programming language version 3.9.16 (Python Software Foundation) using the Pandas package version 1.4.2 (Python Software Foundation). All statistical analyses were performed in the R programming language version 4.2.0 (R Project for Statistical Computing). Cox proportional hazards modeling was done using the coxph function in the survival package version 3.5 (R Project for Statistical Computing), and spline modeling was performed using penalized cubic splines with 2 *df* using the psplines function from the survival package. Graph visualization was performed using Gephi software version 0.10.1 (NetBeans). The health care system with the largest connected component of clinician interaction was selected for visualization.

**Table 1.** Clinicians by Variable and Adoption Status at the End of the Study^a^

Variable	Clinicians, No. (%)		Adopter, %	*P* value
	Nonadopter (n = 800)	Adopter (n = 227)		
Clinician role				
Attending physician	394 (49)	112 (49)	22.1	.90
Resident physician	230 (29)	68 (30)	22.8	
Advanced practice practitioner	175 (22)	47 (21)	21.2	
Health care system				
1	244 (31)	13 (6)	5.1	<.001
2	174 (22)	18 (8)	9.4	
3	21 (3)	24 (11)	53.3	
4	264 (33)	68 (30)	2.5	
5	96 (12)	104 (46)	52.0	
Site status				
Intervention site	416 (52)	173 (76)	29.4	<.001
Usual care site	383 (48)	54 (24)	12.4	
Exposure status				
Exposed ≥1 times	110 (14)	40 (18)	26.7	.18
Nonexposed	689 (86)	187 (82)	21.3	
No. of exposures percentile				
50th	0	0	NA	NA
95th	1	3	NA	
99th	2	6.7	NA	

Abbreviation: NA, not applicable.

^a^ See time-to-event analysis in the text for a more detailed estimate of associations between covariate value and adoption status.

In sensitivity analyses, conversions and, therefore, exposures before the study start period may have been present but were not measurable, although reported buprenorphine use rates before the study were 0% to 2%.^21^ To perform a sensitivity analysis for unmeasured exposures before the study start, we defined a second outcome variable as the cumulative number of exposures in the prior 4 months, discarding conversions in the first 4 months of the study period and repeating the analysis described above. To perform a sensitivity analysis for unmeasured conversions before the study start period, we performed analyses where the first and last 10%, 20%, and 30% of clinicians who converted were removed from the outcome measure but still allowed to expose other clinicians. This tested bias that might be introduced if these clinicians either prescribed more readily (converting early) or offered the prescribing experience to other clinicians on their team (converting late).

## Results

**Figure 1.**
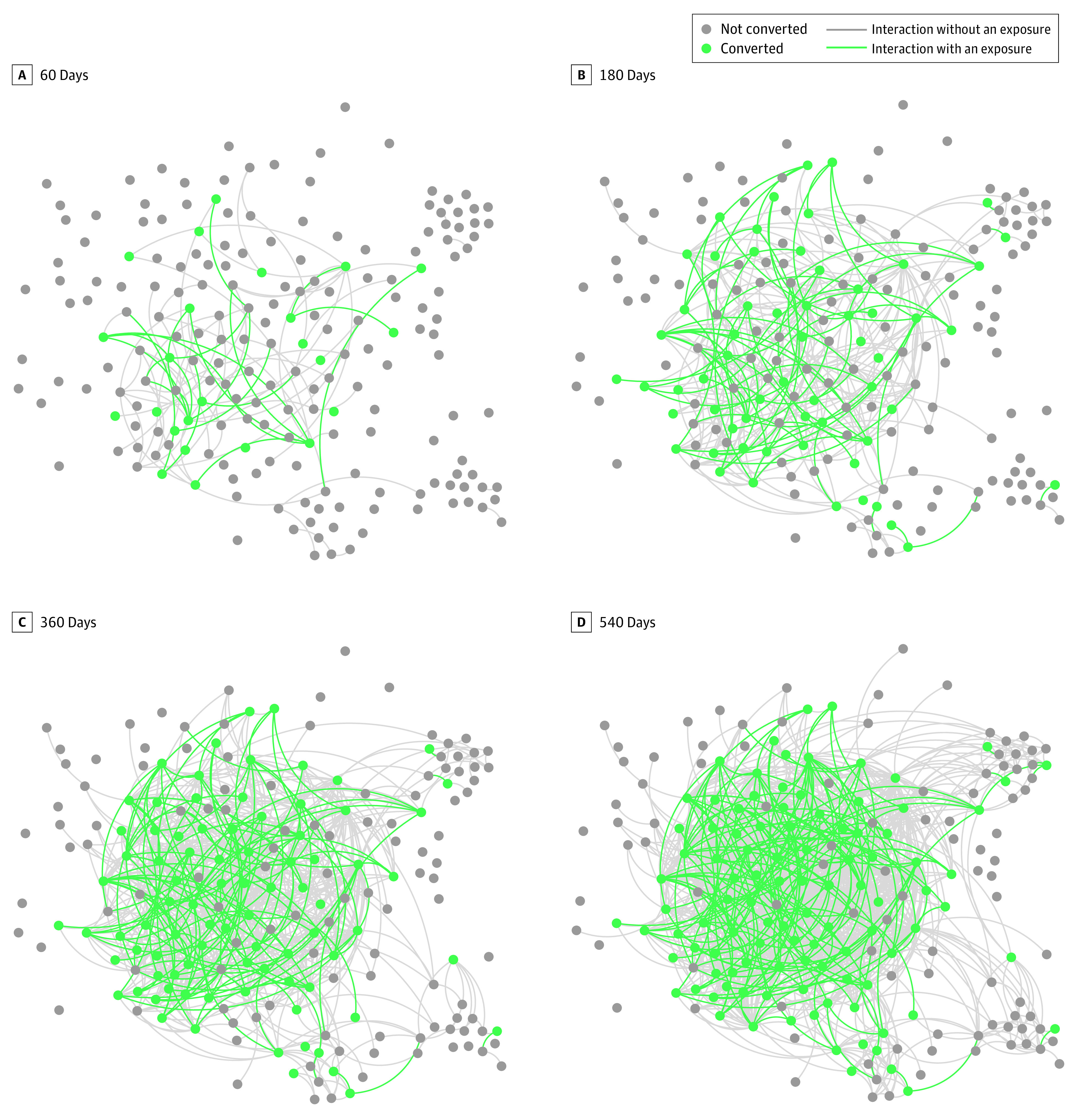
Clinician Interaction, Exposure, and Adoption Network Clinician exposure and conversion network for health care system 5 at 4 time points during the study. Each point represents a clinician, and the color denotes whether the clinician had converted to adopter status by the given time.

There were 7831 ED patient visits that met the criteria for OUD, and 728 (9.3%) of these resulted in an initiation of buprenorphine. The set of all encounters involved 1026 unique clinicians (eFigure 1 in Supplement 2) with 10 240 pairwise interactions among them. Attending physician demographics have previously been reported.^22^ Demographic data on resident physicians and APPs were not available because of limitations of identifiable data collection that were prespecified in the trial protocol due to the waiver of informed consent. Of these interactions, 713 (7% of total pairwise interactions) represented exposure events in which a clinician who had not yet initiated buprenorphine was on a care team with another clinician who initiated buprenorphine. At the completion of the study, 227 clinicians (22%) had initiated buprenorphine at least once, and 150 (14.6%) were exposed at least once. The distribution of conversion status at study completion stratified across categories of clinician role, health system, intervention site status, and exposure status is shown in Table 1. Four time points in the dynamic clinician interaction network showing exposures and adoption status for the health care system with the largest connected component of clinician interactions are shown in Figure 1.

**Figure 2.**
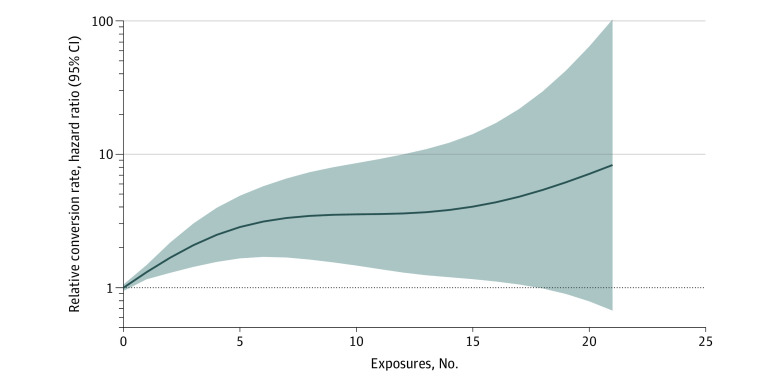
Association of Conversion to Buprenorphine Prescriber Status With Cumulative Number of Exposures to Buprenorphine Prescribing The solid line shows the estimated relative conversion rate, and the shaded area denotes the 95% CI.

Time-to-event modeling of first buprenorphine initiation using Cox proportional hazards was performed to examine factors associated with clinician adoption of the practice of ED initiation of buprenorphine. We adjusted for clinician type owing to training and regulatory differences by role (eg, the requirement for X-waiver training). Compared with APPs, attending physicians and resident physicians had a lower hazard ratio (HR) for converting to prescriber status (attending physicians, HR, 0.62; 95% CI, 0.43-0.90; resident physicians, HR, 0.52; 95% CI, 0.35-0.78). Intervention site status was also associated with higher rates of adoption (HR, 1.50; 95% CI, 1.04-2.18). The health care system where the clinician worked was significantly associated with the hazard of conversion. Health care system 1 had lower rates of buprenorphine prescribing than all other sites within the study (Table 2). Cumulative number of exposures was associated with an overall increase in hazard of conversion with a significant nonlinear effect estimated using penalized splines (Table 2). The estimated hazard as a function of the cumulative number of exposures is shown in Figure 2. Conversion rates are relative to the rate of conversion at 0 exposures. The association increased in a dose-dependent manner (1 exposure: 1.31; 95% CI, 1.16-1.48; 5 exposures: HR, 2.85; 95% CI, 1.66-4.89; 10 exposures: HR, 3.55; 95% CI, 1.47-8.58). Estimates for higher exposure values show increased variance in concordance with the smaller number of clinicians with high number of exposures who had not already initiated buprenorphine.

**Table 2.** Cox Proportional Hazards Model of Prescriber Conversion^a^

Variable	HR (95% CI)	*P* value
Clinician role (vs advanced practice practitioner)		
Attending physician	0.62 (0.43-0.90)	.01
Resident physician	0.52 (0.35-0.78)	.001
Intervention site status (vs usual care)	1.50 (1.04-2.18)	.03
Academic site status (vs nonacademic)	3.15 (2.16-4.60)	<.001
Health care system (vs system 1)		
2	3.21 (1.53-6.72)	.003
3	31.50 (15.30-64.50)	<.001
4	5.50 (3.00-10.10)	<.001
5	13.30 (7.32-24.00)	<.001

Abbreviation: HR, hazard ratio.

^a^ HRs were calculated with exposure term fit with penalized cubic spline with 2 *df*. See Figure 2 for values and 95% CIs.

The mean number of other clinicians exposed by an index clinician who adopted the practice was 1.82 (2.5th percentile, 1; 97.5th percentile, 5). eFigure 2 in Supplement 2 shows the number of other clinicians that each index clinician exposed and the number of these other clinicians who subsequently converted. The number of other clinicians exposed by an index clinician ranged from 1 to 13, and the number of these other clinicians who subsequently convert ranged from 0 to 10. After initial conversion, 49.8% of clinicians (113 clinicians) had a subsequent ordering or prescribing event during the remainder of the study period. The distribution of subsequent prescribing rates is shown in eFigure 3 in Supplement 2.

In sensitivity analyses, time-to-event modeling using the number of exposures in the prior 4 months instead of total prior exposures showed a similar dose-dependent association (eFigure 4 in Supplement 2), as well as similar model coefficients (eTable in Supplement 2). Sensitivity analyses to examine the impact of unobserved adoption before the start of the study found a dose-dependent association between exposures and conversion, consistent with the primary analysis (eFigure 5 in Supplement 2).

## Discussion

In this secondary analysis of EMBED trial data on ED initiation of buprenorphine in the setting of user-center CDS, exposure to other physicians who had initiated buprenorphine during the trial was associated with self-adoption of the practice, consistent with a social contagion effect. This was nonlinear, with subsequent exposures having a greater estimated HR than initial ones. These findings were present after controlling for associated factors, including health care system and clinician role. Health care systems showed substantial variation in HR for adoption (Table 2), and APPs were more likely to convert to prescribing buprenorphine than attending physicians.

The factors identified as contributors to ED initiation of buprenorphine in this study extend prior qualitative research focused on barriers to buprenorphine prescribing. Previous survey and interview studies^9,10,11^ have identified lack of training or experience, local cultural factors, and general logistical issues as barriers to buprenorphine prescription. A qualitative study^25^ of physicians in the EMBED trial found that clinicians reported organizational culture, clinician training, and connection to continued treatment as themes that impacted prescribing. Social effects were qualitatively described, with clinicians reporting that a local culture of prescribing or specific local champions facilitated their own prescribing of buprenorphine. Our findings are commensurate with the findings of these prior studies and may help to provide context as to the relative magnitude of each. Health care system had the largest association with HR, suggesting major contributions from systemic processes. Systemic factors may be cultural, related to attitudes or behaviors, or logistical, reflecting challenges in the steps required to prescribe buprenorphine including access to follow-up care. The HR of 1.50 for intervention site status suggests that the logistics addressed by the EMBED intervention may remove some of the barriers to practice adoption across systems, but site-specific systemic barriers may remain, leading to high variation among health care systems. Our intervention site status finding is in agreement with the primary analysis of the EMBED trial,^22^ which found an odds ratio of 1.83 for attending physicians initiating buprenorphine at least once between control and intervention sites. Connection to community referral, which was limited at some sites within the EMBED trial, may represent 1 type of systemic barrier contributing to this variation among sites. The association of clinician role with adoption rates may reflect experiential differences, willingness to change historical practice patterns, or key logistical barriers because of the X-waiver requirement for outpatient buprenorphine prescribing during the trial, which may be possessed in lower proportion by residents compared with advanced practice registered nurses (part of the APP subgroup).^26^ The recent federal changes to the X-waiver requirement, therefore, may represent a step in minimizing this differential adoption rate.^27,28^

This study also extends prior quantitative work examining the uptake of practice after an intervention by using a time-to-event framework instead of reporting prevalence of the practice, as well as adjusting for health care system characteristics, physician characteristics, social connections, and study duration.^22^ The time-to-event framework was used to capture the clinician interaction dynamics to determine whether evidence for social contagion was present. The association with cumulative exposure and practice adoption may reflect aspects of social contagion leading to mitigation of these barriers. By seeing examples of buprenorphine initiation, those on the care team who did not prescribe may gain comfort or skills that make self-adoption of the practice more likely.^9^ The nonlinearity of the association between cumulative exposure and practice adoption may represent a dose-response association for the level of experience required for adoption. Further research using mixed methods to assess whether attitudes and comfort changes in parallel with the number of observed prescribing encounters could help elucidate the mechanism. Additional work could prospectively study or leverage this identified dose-response association. An important consideration of practice adoption is whether the practice is maintained after it is initially performed. We found that approximately one-half of adopters have a subsequent order or prescription within the study period. Future work with longer follow-up is needed to understand the factors contributing to practice consistency.

### Limitations

This study has several limitations. First, data on which clinicians had ever prescribed buprenorphine before the study start date are unknown. The adoption end point is, therefore, best interpreted as time-to-next-event analysis from the study start date, and the factors presented here represent HRs for this event as well as demonstrating an additive effect of the trial intervention on contagion of events. This may affect the social contagion analysis by hiding exposures that occurred between adopters before the study start date. The potential effect of these unknown data on the current study is tempered by data showing that the reported rates of buprenorphine use were low across all sites, at 0% to 2% per encounter with OUD, with most sites reporting 0% use.^21^ Second, buprenorphine initiation requires the appropriate clinical context, and the rate of encounters meeting these requirements may be different for different adopters. To adjust for this, we included the health care system as a covariate hypothesizing encounter rates should be similar for physicians working within the same EDs. Subsequent analyses could focus on parameterizing rate of conversion on the number of potential encounters in which buprenorphine is initiated, rather than elapsed time. Third, in the time-to-event analysis, we consider all exposures to be of equal weight, but prior literature shows that individuals, labeled as local champions, can have a larger impact on those around them.^25^ We see evidence for heterogeneous effects of exposures based on the range of conversion rates shown in eFigure 5 in Supplement 2. This highlights the need for future research to study factors of exposures that lead to effective conversion. Fourth, buprenorphine ordering for ED administration, prescribing for outpatient administration, and authorization of these orders are grouped in the main outcome but may represent heterogeneous outcomes because of the presence of X-waiver requirements for outpatient prescriptions. To address this, we have included the clinician type as a covariate for adjustment, but future work examining differences between administration and outpatient prescription is necessary. Fifth, APPs may have ordered or prescribed without attending physician authorization but with attending physician approval. This approval would be captured as an exposure for the attending physician but not a conversion, since the attending physician did not perform one of the outcome event actions. This may be an explanation for the lower rate of conversion of attending physicians compared with APPs, who may order and prescribe in conversation with attending physicians. This potential bias may be tempered by the fact that number of encounters for patients with OUD exceeds those where buprenorphine is ordered or prescribed, which represent untaken opportunities for clinicians to adopt the practice. Sixth, although infrequent, clinicians occasionally worked at multiple sites that spanned different categorical variables. This crossover may have led to spread that was not fully characterized in the present study.

## Conclusions

A challenge facing clinicians in confronting the opioid crisis is how to close treatment gaps including adopting the practice of buprenorphine initiation. Here, we identify that although health care system (and associated system-level factors) is a major factor associated with buprenorphine initiation, these factors may be modifiable through an intervention such as the EMBED CDS tool, as well as social factors relating to observing colleagues adopting a practice. The remaining variability between adoption at a health care system level shows a need for additional efforts to identify barriers to buprenorphine initiation and interventions to target them. These may include robust connection to outpatient clinicians and interventions targeted to change local culture. Furthermore, changes to the X-waiver requirement may impact these barriers. This study identifies not only a role for both top-down systemic efforts to impact buprenorphine initiation rates but how individual clinicians may be able to affect the practice of those around them.

## Notes

Supplement 1.
Trial Protocol and Statistical Analysis Plan

Supplement 2. **eTable.** Cox Proportional Hazards Model of Prescriber Conversion Based on Prior 4-Month Exposure Count^a^ **eFigure 1.** Study Flow Diagram **eFigure 2.** Conversion of Others Exposed by an Index Clinician **eFigure 3.** Rates of Subsequent Prescribing or Ordering Events **eFigure 4.** Association Between Conversion to Buprenorphine Prescriber Status and Number of Exposures to Buprenorphine Prescribing Within the Last Four Months **eFigure 5.** Sensitivity Analysis: Assessing Bias Introduced by Clinicians Who Converted Prior to the Study Period

Supplement 3.
Data Sharing Statement
