# Supplementary material for: Adoption of Emergency Department–Initiated Buprenorphine for Patients With Opioid Use Disorder: Secondary Analysis of a Cluster Randomized Trial
Source: JAMA Netw Open. 2023 Nov 10;6(11):e2342786. doi: 10.1001/jamanetworkopen.2023.42786 (PMC10638655; doi:10.1001/jamanetworkopen.2023.42786)
Supplement: Supplement 1. — Trial Protocol and Statistical Analysis Plan [file jamanetwopen-e2342786-s001.pdf]

IRB Approved at the
Protocol Level
Dec 06, 2018

User-Centered Clinical Decision Support to Implement Emergency Department-Initiated Buprenorphine for Opioid Use Disorder: Protocol for the Pragmatic Group Randomized EMBED Trial

ABSTRACT

Introduction

Emergency Departments (EDs) frequently care for individuals with Opioid Use Disorder (OUD). Buprenorphine (BUP) is an effective treatment option for patients with OUD that can safely be initiated in the ED. At present, BUP is rarely initiated as a part of routine ED care. Clinical decision support (CDS) represents a potential approach to accelerate adoption of this best practice into routine emergency care. The goal of this trial is to determine whether implementation of a user-centered clinical decision support (CDS) system can increase adoption of initiation of BUP into the routine emergency care of individuals with OUD.

Methods

A pragmatic cluster randomized trial is planned to be carried out in 20 EDs across six healthcare systems over 18 months. The study will include clinicians practicing in these EDs who care for adult ED patients with an electronic health record (EHR) phenotype consistent with OUD not currently taking medication for OUD (MOUD) treatment. The intervention will consist of a user-centered CDS integrated into ED clinician electronic workflow and available for guidance to: 1) determine whether patients presenting to the ED meet criteria for OUD, 2) assess withdrawal symptoms, and 3) ascertain and motivate patient willingness to initiate MOUD treatment. The CDS guides the ED clinician to initiate MOUD treatment and to facilitate follow up as deemed clinically appropriate. Data will be collected pragmatically from the EHR and the CDS web application. The primary outcome is the rate of BUP initiated in the ED (either as administration of BUP in the ED and/or prescription of BUP upon ED discharge). Secondary outcomes are: 1) rates of receiving a referral appointment for addiction treatment, 2) clinician fidelity with the CDS intervention based on a critical action checklist, 3) rates of clinicians providing any ED-initiated BUP, 4) rates of clinicians providing referral for ongoing MOUD treatment, and 5) rates of clinicians who have received Drug Addiction Act of 2000 training. Primary and secondary outcomes will be analyzed using generalized linear mixed models, with fixed effects for intervention status (CDS vs. usual care) and pre-specified site and patient characteristics and random effects for study site.

Ethics and Dissemination

The study is under review with a central IRB. The local IRBs at each participating sites will implement reliance agreements with the central board. No identifiable private information will be collected from patients, thus the patients are not considered human subjects. A waiver of informed consent will be obtained for data collection of clinicians. As a minimal risk implementation study of established best practices, an Independent Study Monitor will be utilized in place of a formal Data Safety Monitoring Board (DSMB). Results will be published in open-access, peer-reviewed journals, presented at national meetings, and shared with the clinicians at participating sites via a broadcast e-mail notification of publications.

Trial registration number: NCT03658642

INTRODUCTION

Background & Rationale

Dependence on opioids is a major public health problem in the United States, taking a devastating toll on Americans, their families, and communities.[1,2] An estimated 2.1 million people in the U.S. have opioid use disorder (OUD)[3] and more than 33,000 opioid-related deaths occur annually.[4] In 2011, there were 605,000 ED visits related to opioids in the United States.[5] From 2016-2017, emergency departments (EDs) experienced a 30% increase in visits for opioid overdose.[6] The ED offers a unique treatment opportunity for patients receiving care for acute and comorbid conditions related to opioid use.

One of the most promising treatments for OUD is buprenorphine/naloxone (BUP), a partial opioid agonist combined with an antagonist, that can be prescribed by an appropriately trained clinician in an office setting for use at home. BUP decreases mortality as well as symptoms of withdrawal, craving, and opioid use.[7,8] In a placebo-controlled randomized trial of 40 OUD patients who all received cognitive-behavioral group therapy, weekly individual counseling, and weekly urine drug screening, cumulative retention in treatment at one year was 75% for individuals in the BUP group compared to 0% in the placebo group ($p = 0.0001$).[9] A recent Cochrane review including 31 trials with 5430 participants found high quality evidence that BUP is superior to placebo in retention of participants in treatment and can reduce illicit opioid use effectively compared to placebo.[10]

Currently, ED clinicians often provide OUD patients referral to opioid treatment programs rather than initiating MOUD treatment in the ED. In a randomized clinical trial involving 329 individuals with OUD, we found that ED-initiation of BUP with referral for ongoing MOUD treatment was superior to referral alone, resulting in nearly twice the percentage of patients who were engaged in formal addiction treatment at 30 days (78% with BUP vs 37% with referral alone vs 45% with brief intervention, $p < 0.001$) and less illicit opioid use.[11] Despite the efficacy of ED-initiated BUP with referral for ongoing MOUD treatment, it is currently not offered in routine ED practice due to multiple complex medical, regulatory, and logistical barriers.[11–13] Adopting this evidence-based practice into routine care would shift the clinical practice paradigm for early OUD identification and treatment by initiating treatment at a time when the patient may be motivated and particularly vulnerable to morbidity and mortality.[14,15]

Clinical decision support (CDS), computerized tools that offer patient-specific assessments or recommendations to clinicians, represents one approach to embed this complex intervention into routine emergency care.[16,17] However, CDS faces its own challenges, including unintended consequences such as alert fatigue and increased cognitive load.[18–22] CDS design recommendations suggest careful consideration of the socio-technical environment and delivery of the right information, to the right person, in the right format, at the right time in clinical workflow to optimize medical decision-making.[23–26]

Objectives

For these reasons, we employed a user-centered design process to design and formatively evaluate the EMBED (Emergency Department-Initiated Buprenorphine for Opioid Use Disorder) CDS intervention. The user-centered design and formative evaluation of the EMBED intervention is reported elsewhere. Given the current opioid epidemic in the US, there is great urgency for prospective trials to identify the best approaches to BUP implementation and integration into routine practice. The goal of this multicenter, pragmatic, parallel cluster randomized trial is to compare the effectiveness of user-centered CDS for ED-initiated BUP and referral for ongoing MOUD treatment to usual care on the rates of ED initiation of BUP and referral in ED patients with OUD. We hypothesize that rates of ED-initiation of BUP and referral will be higher in the user-centered CDS arm of the trial.

Study Design

The study design is an 18-month pragmatic, parallel, cluster randomized, superiority trial using constrained randomization of clusters to arms (schematic diagram, **Figure 1**).[27–29] The unit of randomization (i.e. cluster) is the ED. EDs will be randomly allocated with an allocation ratio of 1:1. Adequate lead time will be allotted to install the intervention in the EHR at all intervention sites--including a three month implementation and washout phase. The intervention will then begin at the same time across all sites with the CDS intervention fully implemented in the intervention sites' EHRs at the start of the trial. Clinicians at control sites will retain all control of their practice and practice as usual without the CDS intervention installed in their EHR.

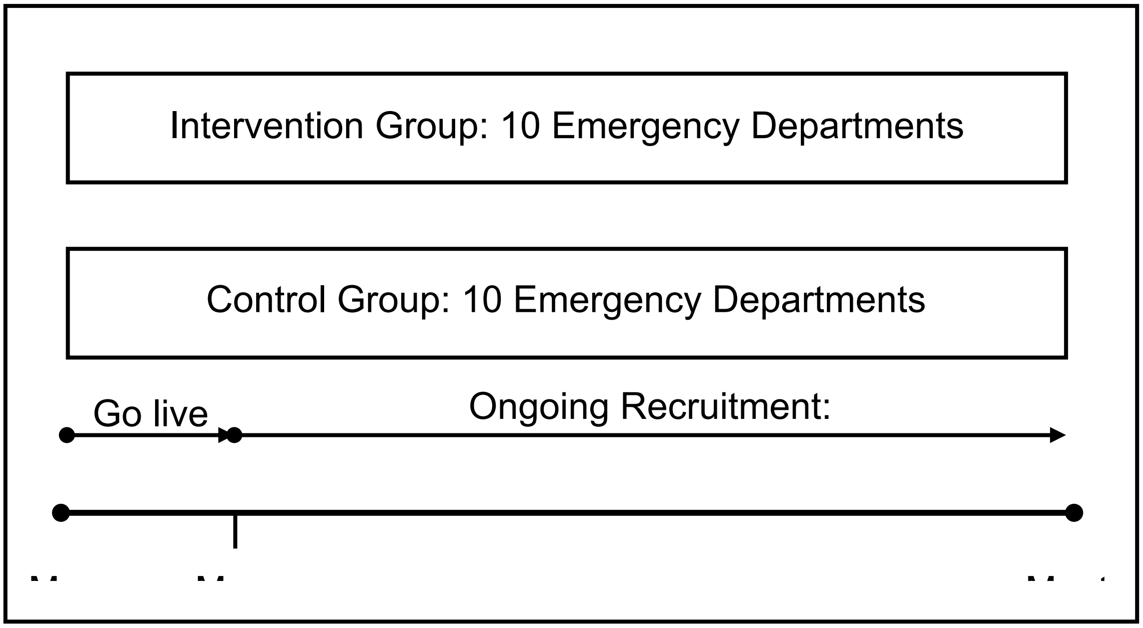

Pragmatic trials study an intervention under the usual conditions in which it will be applied; as opposed to an explanatory trial which would test an intervention under ideal conditions.[27,30] In cluster randomized trials, treatment intervention is allocated to clusters (i.e. groups of individuals) rather than individuals. This is done to manipulate the physical or social environment of the intervention when an individual intervention would likely result in contamination between intervention and control participants at the group level.[28] The parallel cluster randomized design was chosen over a stepped wedge design due to the high likelihood of confounding by temporal trends from ongoing efforts to mitigate the opioid epidemic.[31][32] A major challenge of the cluster randomized design is from potential confounding due to a limited number of heterogeneous groups.[28] Constrained randomization offers a solution to this source of confounding by balancing key cluster-level prognostic factors across the study to avoid distorting estimates of treatment effect due to the confounding factors.[29] This allocation technique more evenly distributes potential confounders between intervention arms by specifying the confounding factors, characterizing each cluster in terms of these factors, identifying a subset of randomization combinations of clusters that adequately balance confounding factors between intervention arms and randomly selecting one of these combinations as the allocation scheme.[29] Potential confounders that will be used for this trial are: EHR vendor, ED annual volume, ED type (e.g., academic, community, urban, rural, etc), ratio of ED clinicians who have a waiver to prescribe BUP, current rate of ED BUP prescribing, resources in ED to facilitate management of patients with OUD, and willingness of staff to adopt the practice of ED-initiation of BUP.

METHODS

Participants

There will be 20 participating EDs from hospitals within approximately six health care systems (HCS). At the time of writing this protocol, all of the sites have very low (or 0) rates of BUP initiation in the ED. The final study sites will be determined based on sample size needs, anticipated number of eligible patients per site determined by electronic health records (EHR) phenotype,[33–36] and willingness and ability to participate (e.g., EHR integration of the intervention, EHR data extraction, availability of BUP in the ED and referral for ongoing MOUD treatment in the surrounding community). When finalized, the full study site list will be available at clinicaltrials.gov.

The intervention will be conducted at the site level. Patients are not considered human subjects since: (1) no identifiable private information will be collected, (2) the intervention does not target the patient, and (3) EHR data will be collected retrospectively without interaction with the patient. The study sample will include all ED clinicians credentialed to practice in the study site EDs. Clinicians who practice at both an intervention and control site will be analyzed with the intervention group. Patients under these clinicians' care at a control site will be excluded from the primary analysis.

Adult ED patients (age 18 years or older) meeting an EHR-derived phenotype suggesting possible OUD will be included in the analysis: those who are discharged from the ED, not pregnant, and not currently taking a MOUD. The CDS will also be available for physicians to use when patients do not meet the EHR phenotype. The initial phenotype has been developed by the study team and is currently undergoing validation via emergency physician chart review to determine the phenotype's validity in identifying the target patient population.[33] Details of this phenotype and its validation will be reported separately. All ED patients meeting the EHR phenotype criteria will be eligible for the trial. For patients with more than one ED visit during the study period, only the initial ED visit will be eligible for inclusion in the primary analysis. The CDS will also be available for clinicians to use for patients who are not identified by the phenotype. These patients will be excluded from the primary analyses.

Intervention

The intervention for this study includes the user-centered CDS as well as education of ED clinicians practicing at all study sites.

The need for flexibility in the graphical user interface of the intervention resulted in the decision to develop the CDS as a web application. This provides the ability to access the tool both embedded within the EHR or directly over the Internet. The web application was developed as a single-page application (SPA) based on React JavaScript library. The CDS is a user-initiated, SMART on FHIR (Substitutable Medical Applications and Reusable Technologies on Fast Health Interoperability Resources)[37] application that streamlines a flow diagram of our clinical protocol for ED-initiated BUP (**Figure 2)**.

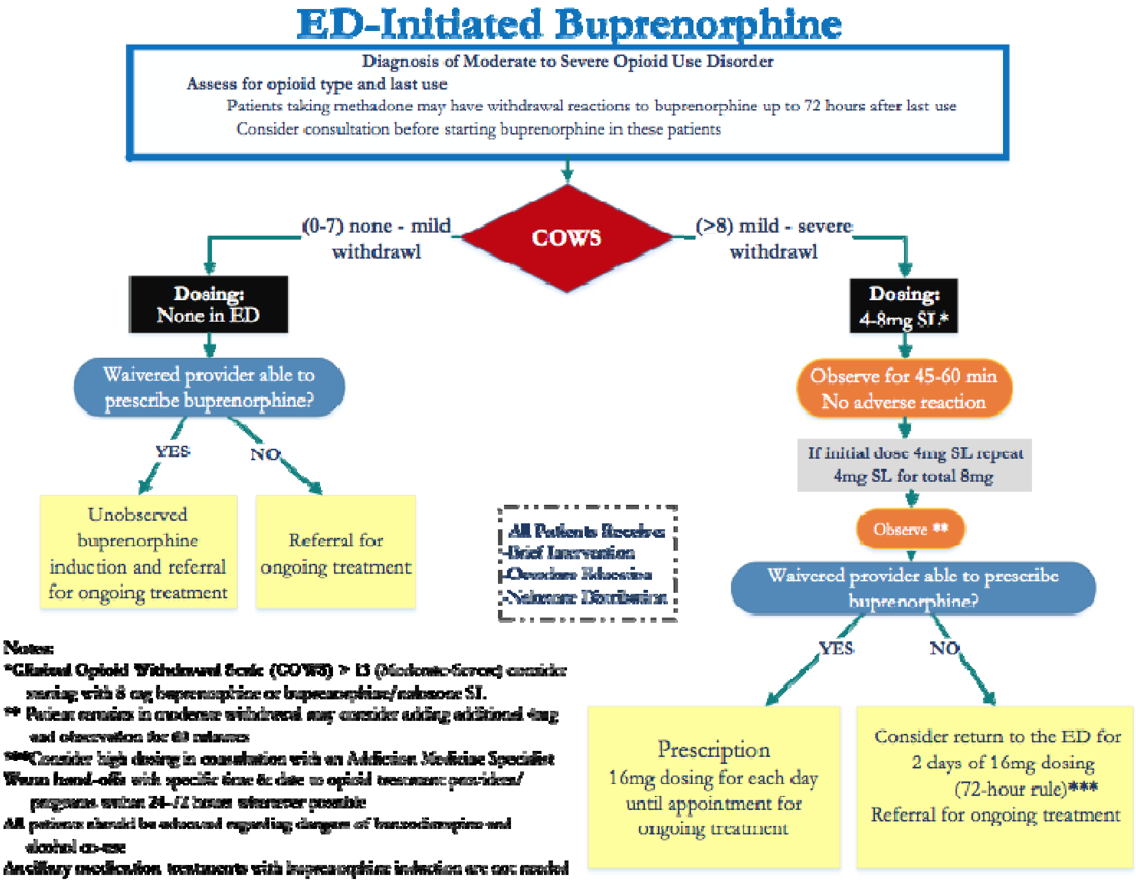

The intervention's graphical user interface (**Figure 3**) is an intuitive, simple layout presenting four care pathways in columns based on the patient's diagnosis of OUD, the severity of withdrawal, and readiness to start treatment. There is additional, optional decision support available for guidance to: 1) evaluate OUD severity based on DSM-5 criteria, 2) assess withdrawal severity using the clinical opiate withdrawal scale (COWS) score, and 3) motivate

patient willingness and readiness to initiate MOUD treatment with a brief motivational interview.[38,39] These materials are also available to share with other members of the care team via a web address, text messaging, or QR code. The interface also includes a toggle switch for the user based on whether or not they have a waiver to prescribe BUP. Non-waivered clinicians cannot prescribe BUP but can administer a one-time dose of BUP in the ED for up to 72-hours.[40] When integrated into the local EHR system, launching a care pathway enables the user to: place orders, refer for ongoing MOUD treatment, and update clinical notes.

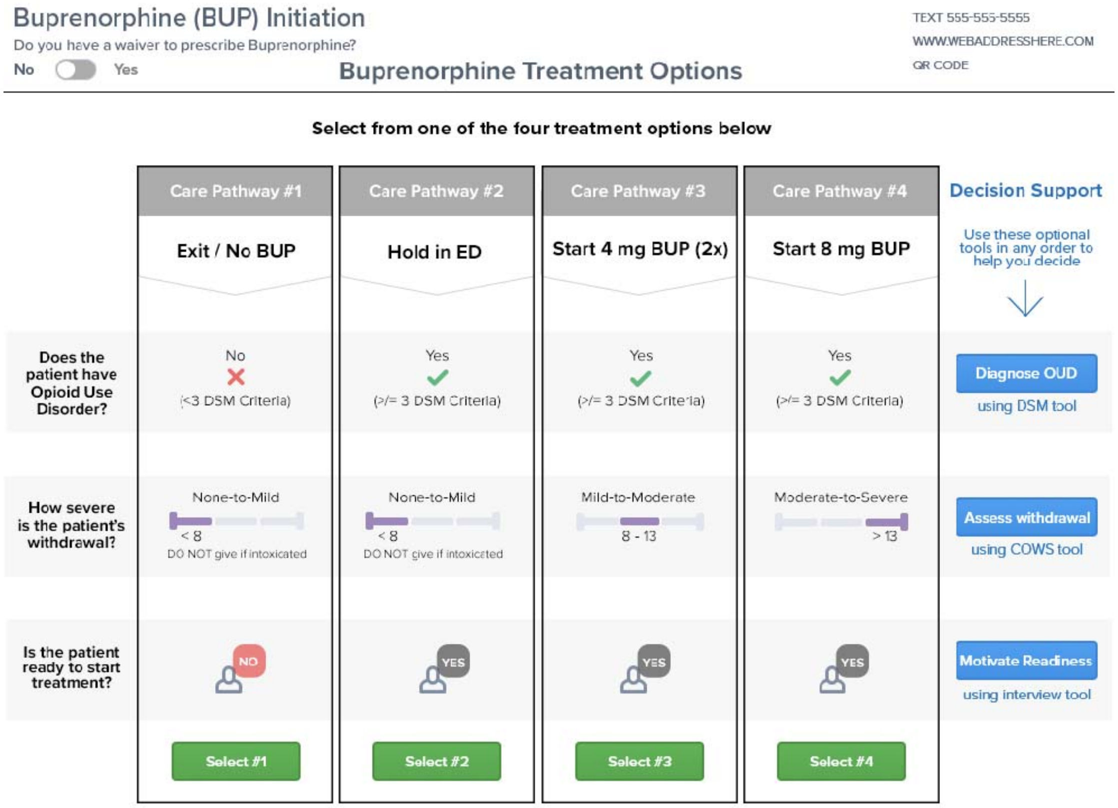

The educational plan will be site-specific and tailored to the usual care at that institution. It will be administered within three months of the study start date. The details of the plan will be developed in partnership with local champions who self-identify an interest in helping to implement an ED-initiated BUP protocol at their site. Specifically, the education plan will be required to include:

1. A didactic on opioid use disorder, its diagnosis, assessment of withdrawal severity, and local resources for referral for ongoing MOUD treatment
2. Circulation and posting in each study site ED of the flow diagram of the study's clinical protocol for ED-initiated BUP (**Figure 2**). Since this protocol is considered best practice,

clinicians at control sites will retain all control of their practice and be encouraged to follow this protocol even though the CDS will not be available to them.

3. Intervention sites will include strategies to increase use of the intervention by training clinicians on how to launch and use the CDS. Use of the intervention will be tracked with site-specific audit and feedback that is consistent with typical quality improvement initiatives at that site.

Given the ongoing and escalating opioid epidemic and wide scope of this trial, we anticipate that there may be concomitant interventions to stem OUD at study sites during the trial. We plan to permit these interventions as long as they are: (1) implemented before randomization so that they can be tracked and accounted for in the constrained randomization process, and (2) they are not a health IT intervention targeted at clinicians to initiate BUP in the ED.

Outcomes

The primary study hypothesis is that there will be higher rates of provision of ED-initiated BUP with referral for ongoing MOUD with user-centered CDS compared with usual care. Therefore, the primary outcome will be BUP initiation in the ED, defined as whether or not an eligible patient is administered BUP in the ED and/or prescribed BUP upon discharge from the ED. Although this is not a patient-centered outcome, it is a pragmatic and meaningful surrogate that will serve as a lead indicator of the CDS intervention's effect on engaging more OUD patients in treatment.

We will also evaluate the effect of user-centered CDS on the following secondary implementation outcomes as compared to usual care, including several following the RE-AIM framework:[41,42]

1. Referral to follow-up for ongoing MOUD treatment (patient-level; Y/N)treatment
2. Prescription for naloxone at ED discharge (patient-level; Y/N)
3. Receipt of discharge instructions on opioid use, overdose education, naloxone education, and buprenorphine education (patient-level; Y/N)
4. Clinician adoption rates (clinician level):
   a. Provision of any ED-initiated BUP during the trial (Y/N)
   b. Provision of any referral for ongoing MOUD treatment during the trial (Y/N)
5. Receipt of Drug Addiction Treatment Act of 2000 training during trial (clinician level; Y/N)

Additional secondary implementation outcomes to be obtained from the web application include: clinician fidelity with the intervention assessed via a critical action checklist[43] and error rate of the intervention (using surrogates based on tool usage, e.g., application launched but not used, launching a page in the web application and spending less than two seconds on that page). The intervention will continue to be made available for use after the trial concludes; three months after trial completion, medical record review of eligible patients will be conducted at a subset of intervention sites to determine the maintenance rate of the intervention.

Sample Size

Current rates of BUP use in the ED range from 0-2% with most sites at 0%. Assuming a rate of BUP use in the usual care group of 1%, an increase to 10% would be a convincing and meaningful incremental effect of the intervention. Preliminary data from EDs that will be randomized in this trial suggest an Intraclass Correlation (ICC) for BUP use of 0.01. The NIH group randomized sample size calculator[44] was used to determine the required number of sites to be randomized. With a two-sided type I error of 0.05, a conservative ICC of 0.03, and an expected average of 200 participants per site a total of 12 sites will provide 90% power to detect a difference of 9%. This estimate is based on the assumption that all sites will have 200 unique patient visits during the trial that meet the EHR phenotype.

We evaluated the impact of enrollment variability across sites on the required sample size using the formula described by Eldridge et al.[45] We added 2 sites to the total number of sites given the use of z-scores rather than t-scores in the estimation. As the coefficient of variation (CV) in the number of participants enrolled across sites increases, the required number of sites increases (**Figure 4**). We will randomize a total of 20 sites to accommodate this potential variability.

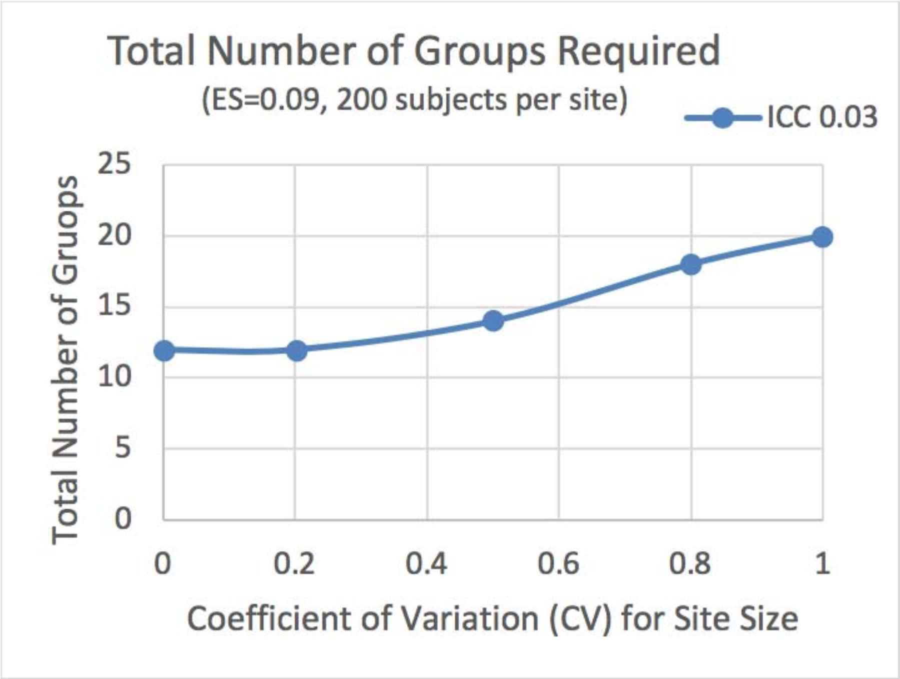

Table 1 shows the power to detect different effect sizes given randomization of 20 sites. Even with large variability in participant enrollment (CV=1), we will have over 90% power to detect a difference of 0.09. We will have good power (>80%) to detect effect sizes as low as 0.05 provided the variability in site enrollment is not great (<0.50).

		**Effect Size (Difference in Proportions)**				
**Coefficient of Variation in Enrollment**		**0.05**	**0.06**	**0.07**	**0.08**	**0.09**
	**0**	87%	94%	97%	99%	99%
	**0.2**	86%	93%	97%	99%	99%
	**0.5**	80%	89%	94%	97%	98%
	**0.8**	70%	80%	87%	92%	95%

	**1.0**	62%	72%	80%	87%	91%

Allocation

Potential study sites that meet readiness criteria at the time of randomization will be allocated 1:1 to CDS and usual care groups using constrained randomization conducted by personnel in the data coordinating center (DCC). The general method will follow procedures and recommendations from the literature on group randomized trials.[29]

With a small number of sites that differ in important ways, unconstrained randomization may not adequately balance important site characteristics. To improve comparability of treatment and control sites, personnel in the DCC under the direction of JDD will list all possible allocations of treatment and control groups (with 20 sites, there are about 165,000 combinations of treatment and control groups). The imbalance score (□) from Raab and Butcher will be calculated for each possible allocation.[46]

$$\beta = \sum_{l=1}^{S} \omega_l (\bar{x}_{0l} - \bar{x}_{1l})^2$$

where *S* is the number of variables on which the groups should be balanced, ω_l is a weight calculated as the inverse variance of the mean of variable *l* across the hospitals, and the $\bar{x}_l$ represent the means of variable *l* across the hospitals in the intervention (indexed as 1) and control (indexed as 0) groups. A candidate set of 1000 possible allocations with the most favorable imbalance scores will be selected, and the final allocation selected at random from that candidate set.

Since clinicians must know how to launch and use the intervention, they will not be blinded to the allocation of their site as a control or intervention site. Clinicians may inform patients that they are using the CDS or not, as they deem appropriate consistent with CDS use in their usual practice. All study sites will post information in their ED informing patients of the study.

Data Collection

Outcome data will be collected via SQL query of the local EHR at regular intervals from data routinely collected in each hospital's EHR. This will facilitate large-scale data collection that would not otherwise be practical in an explanatory trial.

To enable consistent EHR data collection across sites, a master data dictionary of all data elements will be created. At each study site, the variables in the data dictionary will be validated against the institutional EHR to ensure that the variables are correctly mapped to the EHR field that corresponds to the clinical intent of the variable after accounting for documentation practices and workflow at each site.[47] In particular, the outcome variables of BUP initiated in the ED and referral made for ongoing MOUD treatment will be validated against the EHR to ensure accuracy. For data quality assurance, the mapped variables will be validated against the EHR to ensure that the data are clinically relevant to the goals of the project and correctly represents the clinical data that clinicians use to make decisions. Additionally, data to determine compliance, use, and fidelity with the CDS intervention that could not be reliably abstracted from the EHR (e.g., DSM-5 OUD score, COWS score) will be abstracted from the web application's use logs. Information on whether the patient attended the referred follow-up visit and whether the patient was prescribed BUP as an outpatient will be abstracted from the EHR if available (e.g., if the patient is seen for follow-up within the same system).

Data will be sent from study sites to the study DCC at predetermined, regular intervals. The DCC will conduct ongoing data monitoring activities on study data from all participating sites to ensure data received is what it is intended to be. Baseline data for the study participants will include demographic and clinical data such as age, gender, race, ethnicity, insurance status, past medical and psychiatric history, recent medical or psychiatric hospital admissions, recent enrollment in formal addiction treatment, active prescriptions for other opioids, and urine drug screen results as ascertained by regularly collected data in the EHR.

Data Management

Study data will only be available to members of the study DCC who are authorized for this study. To ensure the privacy and confidentiality of data for this project, DCC servers hosting data repositories are strongly firewalled; access to the repositories is permitted only through properly authenticated Web APIs. All data is encrypted both at rest and in transit. The DCC database-hosting is certified by the our institution's Information Security Office as conforming to HIPAA and our institution's data protection guidelines. All personnel who have access to the data will pass appropriate HIPAA training coursework. All network communication will transpire via security transport layer for web applications. All project computers are stored in locked offices within a building having limited, electronic passkey access. All computers are password protected and protected by our institution's firewall which is encrypted using Microsoft BitLocker.

Individually identifiable or deducible data will only be by transmitted via secured telecommunications, never by unsecured telecommunications like email or electronic File Transfer Protocol (FTP). Procedures are in place for rapid recovery from hardware or database failure.

Data Monitoring

As a minimal risk implementation study of established best practices, an Independent Study Monitor will be utilized in place of a formal Data Safety Monitoring Board (DSMB). Interim monitoring will focus on adherence to the protocol, completeness of data retrieval from each ED's EHR, and uptake of the CDS intervention. A set of monitoring tables will be generated for this purpose. The Independent Study Monitor will report directly to the study DCC. No interim analyses for effectiveness are planned.

Analysis Plan

*General Considerations***:** This is a cluster randomized trial to test the hypothesis that there will be higher rates of provision of ED-initiated BUP and referral for ongoing MOUD with user-centered CDS compared with usual care. Analyses will be conducted as intention to treat including all individuals regardless of intervention receipt. While the unit of randomization is at the level of the ED, the unit of analysis will be the patient or clinician. Analyses of primary and secondary outcomes will be conducted using generalized linear mixed models (GLMM) to account for clustering from the EDs and clinicians in patient outcome models. [31] Analyses will be performed in SAS v9.4 (Cary, NC) with a two-sided type I error of 0.05 (unless otherwise specified). For the primary and secondary analyses described below, only the first ED encounter for an individual patient will be used. Supportive analyses will include patients with repeated ED visits.

*Comparability of Baseline and Intervention Patients***:** Distributions of baseline demographic and clinical characteristics will be described during baseline and intervention periods. Comparability for continuous variables will be examined graphically and by summary statistics (means, medians, quartiles, etc.). Categorical variables will be examined by calculating frequency distributions.

*Analysis of Primary Outcome***:** The primary outcome, initiation of BUP in the ED, will be assessed for all patients that meet the criteria for the EHR phenotype. Intervention differences

(CDS vs usual care) for this dichotomous outcome will be examined using mixed effect logistic regression (GLMM). This model will contain a fixed effect for intervention (CDS vs usual care). Random effects will be included for ED and the primary clinician to account for clustering of responses. The model will also include cluster-level covariates included in the constrained randomization and patient-level covariates that may be associated with the delivery of BUP (age, gender, race, ethnicity, insurance status, past medical and psychiatric history, recent medical or psychiatric hospital admissions, recent enrollment in formal addiction treatment, active prescriptions for other opioids, and urine drug screen results). Linear contrasts will be used to estimate treatment differences along with 95% confidence intervals in the proportions of ED patients that received BUP in intervention vs. usual care. Given the relative advantages of GLMM and Generalized Estimating Equations, sensitivity analyses will compare treatments using a logistic regression with Generalized Estimating Equations, clustering on ED.

Analysis of Secondary Outcomes: Secondary outcomes such as referral for MOUD appointment, attendance at an MOUD appointment (if available in the EHR), prescription for naloxone at ED discharge and receipt of discharge instructions. will be evaluated using random effects logistic regression as described above. Assessments of the clinician including provision of any ED-initiated BUP during the trial, provision of any referral for ongoing MOUD treatment during the trial and receipt of Drug Addiction Treatment Act of 2000 training during the trial will be compared between intervention and usual care using a GLMM. These models will be stratified by the number of eligible patients the clinician encountered during the trial and will include a fixed effect for intervention, cluster-level covariates included in the constrained randomization, and a random effect for ED. Discrete numeric outcomes such as clinical fidelity will be compared using the GLMM with an log link and a negative binomial distribution.

Plan for Missing Data: Several strategies will be imposed to accommodate the likelihood that missing data will occur during this study. Prevention is the most obvious and effective manner to control bias and loss of power from missing data.[48] As noted in the Data Collection section above, prior to the trial we will pilot data collection procedures. Variables with large proportions of missing will be excluded from collection. We will follow the intent to treat principle, requiring follow-up of all EDs randomized regardless of the treatment received.[49] Regular data retrieval from EHR combined with monitoring and missing data reports will trigger protocols for tracking and obtaining missing data. Despite these prevention efforts it is reasonable to assume missing data will occur. Our primary analysis is valid under the assumption that missing data is missing

at random (MAR).[50] We will evaluate the plausibility of this assumption by determining the extent of missing data and use logistic regression to identify factors associated with missing data. As appropriate, we will conduct sensitivity analysis using pattern-mixture and selection models under missing not at random (MNAR) assumptions to examine the robustness of conclusions of the primary analysis to missing data.[48,50]

ETHICS AND DISSEMINATION

We plan to obtain all necessary regulatory and human subjects protection approvals and procedures including central review board (IRB) approval for all study sites for the conduct of the trial. The local IRBs at each participating site will implement a rely agreement with this central board. We anticipate a waiver of informed consent under the Common Rule (45 code of federal regulations (CFR) 46.116 given that:[51,52] (1) the research involves no more than minimal risk to the subjects;[53] (2) the waiver or alteration will not adversely affect the rights and welfare of the subjects; (3) the research could not practicably be carried out without the waiver or alteration; and (4) subjects will be provided with additional pertinent information after participation.

Patients are not considered human subjects by HHS regulation 45 CFR 46.102(f)[52] since: (1) no identifiable private information will be collected, (2) the intervention does not target the patient, and (3) EHR data will be collected retrospectively without interaction with the patient. Therefore, consent is not applicable to this population. Furthermore, all recommendations included in the CDS intervention are considered best practices in treatment of OUD. The OUD population has a high underlying risk of morbidity/mortality (approximately 5% risk of death in 12 months).[8] Patient rights and welfare will be protected per standard practice. Therefore, the risk to a patient with OUD who is not receiving MOUD treament in their ordinary daily lives greatly exceeds the risk of the EMBED intervention. All study sites will post details about the study in a location visible to patients to make them aware of the option to receive BUP and referral to treatment so as best to offer an informed decision for requesting care. Patients will retain the right to request MOUD treatment at any study site.

Clinicians at all study sites will have access to all standard OUD medications and services to which they would otherwise have access to treat OUD patients. Clinicians will retain all control of their practice and at intervention sites have the option whether or not to use the intervention (i.e., can opt out). Clinician identifiers will be collected in order to follow practice patterns.

However, the investigators will be blinded to both site and clinician identifiers. Each system will use an Honest Broker to protect the welfare and identity of each site and clinician and allow adjudication for analyses. Clinicians will be made aware of the study, its outcomes, the data to be collected and, at intervention sites, how to use and opt out of using the CDS via broadcast e-mail and direct communication by site champions. A flow diagram of the study's clinical protocol (**Figure 2**) will shared with clinicians and posted in the clinical work area of all study sites. Since this protocol is considered best practice, clinicians at control sites will retain all control of their practice and be encouraged to follow this protocol even though the CDS will not be available to them. As this is a pragmatic trial focused on implementing this intervention in a way that is as close to routine care as possible, consenting clinicians would not be consistent with routine CDS implementation and could jeopardize the scientific validity of the CDS intervention to overcome barriers to adoption of this practice[51]. Given the stigma[11] associated with treating individuals with OUD, the additional burden of the consent process could be a deterrent for clinicians to provide MOUD treatment to appropriate patients and bias the sample to clinicians with less stigma toward treating these patients. For this reason and since clinician data will be de-identified and unavailable to the investigators, we propose a waiver of consent of the clinicians to ensure the scientific validity of our findings. There is precedent for such a waiver in a similar situation.[54] Results will be published in open-access, peer-reviewed journals, presented at national meetings, and shared with the clinicians at participating sites via a broadcast e-mail notification of publications.

Because this study uses no investigational agents or devices, and instead uses a health IT intervention to promote recommended clinical practices, an Independent Study Monitor will be utilized in place of a formal DSMB.

REFERENCES

1 Rudd RA, Aleshire N, Zibbell JE, *et al.* Increases in Drug and Opioid Overdose Deaths--United States, 2000-2014. *MMWR Morb Mortal Wkly Rep* 2016;**64**:1378–82.

2 Buchanich JM, Balmert LC, Burke DS. Exponential Growth Of The USA Overdose Epidemic. *bioRxiv* Published Online First: 2017.https://www.biorxiv.org/content/early/2017/05/09/134403.abstract

3 Abuse S. … Services Administration (2017). Key substance use and mental health indicators in the United States: Results from the 2015 National Survey on Drug Use …. *Abuse and Mental Health Services Administration …*

4 Seth P, Scholl L, Rudd RA, *et al.* Overdose Deaths Involving Opioids, Cocaine, and

Psychostimulants - United States, 2015-2016. *MMWR Morb Mortal Wkly Rep* 2018;**67**:349–58.

5 Substance Abuse and Mental Health Services Administration. Drug Abuse Warning Network, 2011: National Estimates of Drug-Related Emergency Department Visits. Published Online First: 5/2013.https://www.samhsa.gov/data/sites/default/files/DAWN2k11ED/DAWN2k11ED/DAWN2k11ED.pdf

6 Vivolo-Kantor AM, Seth P, Gladden RM, *et al.* Vital Signs: Trends in Emergency Department Visits for Suspected Opioid Overdoses - United States, July 2016-September 2017. *MMWR Morb Mortal Wkly Rep* 2018;**67**:279–85.

7 Sullivan LE, Fiellin DA. Narrative review: buprenorphine for opioid-dependent patients in office practice. *Ann Intern Med* 2008;**148**:662–70.

8 Larochelle MR, Bernson D, Land T, *et al.* Medication for Opioid Use Disorder After Nonfatal Opioid Overdose and Association With Mortality: A Cohort Study. *Ann Intern Med* Published Online First: 19 June 2018. doi:10.7326/M17-3107

9 Kakko J, Svanborg KD, Kreek MJ, *et al.* 1-year retention and social function after buprenorphine-assisted relapse prevention treatment for heroin dependence in Sweden: a randomised, placebo-controlled trial. *Lancet* 2003;**361**:662–8.

10 Mattick RP, Breen C, Kimber J, *et al.* Buprenorphine maintenance versus placebo or methadone maintenance for opioid dependence. *Cochrane Database Syst Rev* Published Online First: 2014. doi:10.1002/14651858.cd002207.pub4

11 Sharfstein JM. The Opioid Crisis From Research to Practice. *Milbank Q* 2017;**95**:24–7.

12 We have the tools needed to cut overdose death rates by half — but we aren't using them. Paperpile. https://paperpile.com/app/p/964b2025-4e4d-0756-9b7a-d12f213481d7 (accessed 6 Aug 2018).

13 Walsh L. Buprenorphine Waiver Management | SAMHSA - Substance Abuse and Mental Health Services Administration. https://www.samhsa.gov/programs-campaigns/medication-assisted-treatment/training-materials-resources/buprenorphine-waiver (accessed 17 Jul 2018).

14 Duber HC, Barata IA, Cioè-Peña E, *et al.* Identification, Management, and Transition of Care for Patients With Opioid Use Disorder in the Emergency Department. *Ann Emerg Med* Published Online First: 4 June 2018. doi:10.1016/j.annemergmed.2018.04.007

15 Houry DE, Haegerich TM, Vivolo-Kantor A. Opportunities for Prevention and Intervention of Opioid Overdose in the Emergency Department. *Ann Emerg Med* 2018;**71**:688–90.

16 Kawamoto K, Houlihan CA, Balas EA, *et al.* Improving clinical practice using clinical decision support systems: a systematic review of trials to identify features critical to success. *BMJ* 2005;**330**:765.

17 Garg AX, Adhikari NKJ, McDonald H, *et al.* Effects of computerized clinical decision support systems on practitioner performance and patient outcomes: a systematic review. *JAMA*

2005;**293**:1223–38.

18 Sittig DF, Wright A, Osheroff JA, *et al.* Grand challenges in clinical decision support. *J Biomed Inform* 2008;**41**:387–92.

19 Ash JS, Berg M, Coiera E. Some unintended consequences of information technology in health care: the nature of patient care information system-related errors. *J Am Med Inform Assoc* 2004;**11**:104–12.

20 Ash JS, Sittig DF, Campbell EM, *et al.* Some unintended consequences of clinical decision support systems. *AMIA Annu Symp Proc* 2007;:26–30.

21 Levin S, France DJ, Hemphill R, *et al.* Tracking workload in the emergency department. *Hum Factors* 2006;**48**:526–39.

22 Melnick ER, Nielson JA, Finnell JT, *et al.* Delphi consensus on the feasibility of translating the ACEP clinical policies into computerized clinical decision support. *Ann Emerg Med* 2010;**56**:317–20.

23 Sirajuddin AM, Osheroff JA, Sittig DF, *et al.* Implementation pearls from a new guidebook on improving medication use and outcomes with clinical decision support. Effective CDS is essential for addressing healthcare performance improvement imperatives. *J Healthc Inf Manag* 2009;**23**:38–45.

24 Phansalkar S, Edworthy J, Hellier E, *et al.* A review of human factors principles for the design and implementation of medication safety alerts in clinical information systems. *J Am Med Inform Assoc* 2010;**17**:493–501.

25 Horsky J, Phansalkar S, Desai A, *et al.* Design of decision support interventions for medication prescribing. *Int J Med Inform* 2013;**82**:492–503.

26 Gellert G, Webster S, Gillean J, *et al.* Should US doctors embrace electronic health records? *BMJ* 2017;**356**:j242.

27 Roland M, Torgerson DJ. What are pragmatic trials? *BMJ* 1998;**316**:285.

28 Murray DM, Varnell SP, Blitstein JL. Design and analysis of group-randomized trials: a review of recent methodological developments. *Am J Public Health* 2004;**94**:423–32.

29 Li F, Lokhnygina Y, Murray DM, *et al.* An evaluation of constrained randomization for the design and analysis of group-randomized trials. *Stat Med* 2016;**35**:1565–79.

30 Loudon K, Treweek S, Sullivan F, *et al.* The PRECIS-2 tool: designing trials that are fit for purpose. *BMJ* 2015;**350**:h2147.

31 Hussey MA, Hughes JP. Design and analysis of stepped wedge cluster randomized trials. *Contemp Clin Trials* 2007;**28**:182–91.

32 Hemming K, Haines TP, Chilton PJ, *et al.* The stepped wedge cluster randomised trial: rationale, design, analysis, and reporting. *BMJ* 2015;**350**:h391.

33 Richesson RL, Hammond WE, Nahm M, *et al.* Electronic health records based phenotyping in next-generation clinical trials: a perspective from the NIH Health Care Systems

Collaboratory. *J Am Med Inform Assoc* 2013;**20**:e226–31.

34 Hylan TR, Von Korff M, Saunders K, *et al.* Automated prediction of risk for problem opioid use in a primary care setting. *J Pain* 2015;**16**:380–7.

35 Carrell DS, Cronkite D, Palmer RE, *et al.* Using natural language processing to identify problem usage of prescription opioids. *Int J Med Inform* 2015;**84**:1057–64.

36 Reardon JM, Harmon KJ, Schult GC, *et al.* Use of diagnosis codes for detection of clinically significant opioid poisoning in the emergency department: A retrospective analysis of a surveillance case definition. *BMC Emerg Med* 2016;**16**:11.

37 Mandel JC, Kreda DA, Mandl KD, *et al.* SMART on FHIR: a standards-based, interoperable apps platform for electronic health records. *J Am Med Inform Assoc* 2016;**23**:899–908.

38 American Psychiatric Association. *Diagnostic and Statistical Manual of Mental Disorders (DSM-5®)*. American Psychiatric Pub 2013.

39 Wesson DR, Ling W. The Clinical Opiate Withdrawal Scale (COWS). *J Psychoactive Drugs* 2003;**35**:253–9.

40 Walsh L. Special Circumstances for Providing Buprenorphine | SAMHSA - Substance Abuse and Mental Health Services Administration. https://www.samhsa.gov/programs-campaigns/medication-assisted-treatment/legislation-regulations-guidelines/special (accessed 13 Aug 2018).

41 Glasgow RE, Vogt TM, Boles SM. Evaluating the public health impact of health promotion interventions: the RE-AIM framework. *Am J Public Health* 1999;**89**:1322–7.

42 Glasgow RE, Lichtenstein E, Marcus AC. Why don’t we see more translation of health promotion research to practice? Rethinking the efficacy-to-effectiveness transition. *Am J Public Health* 2003;**93**:1261–7.

43 LeBlanc A, Ruud KL, Branda ME, *et al.* The impact of decision aids to enhance shared decision making for diabetes (the DAD study): protocol of a cluster randomized trial. *BMC Health Serv Res* 2012;**12**:130.

44 National Institutes of Health (NIH). National Institutes of Health (NIH). https://researchmethodsresources.nih.gov/SampleSizeCalculator.aspx (accessed 13 Aug 2018).

45 Eldridge SM, Ashby D, Kerry S. Sample size for cluster randomized trials: effect of coefficient of variation of cluster size and analysis method. *Int J Epidemiol* 2006;**35**:1292–300.

46 Raab GM, Butcher I. Balance in cluster randomized trials. *Stat Med* 2001;**20**:351–65.

47 Gong MN, Schenk L, Gajic O, *et al.* Early intervention of patients at risk for acute respiratory failure and prolonged mechanical ventilation with a checklist aimed at the prevention of organ failure: protocol for a pragmatic stepped-wedged cluster trial of PROOFCheck. *BMJ Open* 2016;**6**:e011347.

48 National Research Council, Division of Behavioral and Social Sciences and Education,

Committee on National Statistics, *et al. The Prevention and Treatment of Missing Data in Clinical Trials*. National Academies Press 2010.

49 Lachin JM. Statistical considerations in the intent-to-treat principle. *Control Clin Trials* 2000;**21**:167–89.

50 Molenberghs G, Thijs H, Jansen I, *et al.* Analyzing incomplete longitudinal clinical trial data. *Biostatistics* 2004;**5**:445–64.

51 McKinney RE Jr, Beskow LM, Ford DE, *et al.* Use of altered informed consent in pragmatic clinical research. *Clin Trials* 2015;**12**:494–502.

52 Office for Human Research Protections (OHRP). 45 CFR 46. HHS.gov. 2016.https://www.hhs.gov/ohrp/regulations-and-policy/regulations/45-cfr-46/index.html (accessed 16 Aug 2018).

53 Lantos JD, Wendler D, Septimus E, *et al.* Considerations in the evaluation and determination of minimal risk in pragmatic clinical trials. *Clin Trials* 2015;**12**:485–93.

54 Suffoletto B, Lynch M, Pacella CB, *et al.* The Effect of a Statewide Mandatory Prescription Drug Monitoring Program on Opioid Prescribing by Emergency Medicine Providers Across 15 Hospitals in a Single Health System. *J Pain* 2018;**19**:430–8.
