# Supplementary material for: Adoption of Emergency Department–Initiated Buprenorphine for Patients With Opioid Use Disorder: Secondary Analysis of a Cluster Randomized Trial
Source: JAMA Netw Open. 2023 Nov 10;6(11):e2342786. doi: 10.1001/jamanetworkopen.2023.42786 (PMC10638655; doi:10.1001/jamanetworkopen.2023.42786)
Supplement: Supplement 2. — eTable. Cox Proportional Hazards Model of Prescriber Conversion Based on Prior 4-Month Exposure Counta eFigure 1. Study Flow Diagram eFigure 2. Conversion of Others Exposed by an Index Clinician eFigure 3. Rates of Subsequent Prescribing or Ordering Events eFigure 4. Association Between Conversion to Buprenorphine Prescriber Status and Number of Exposures to Buprenorphine Prescribing Within the Last Four Months eFigure 5. Sensitivity Analysis: Assessing Bias Introduced by Clinicians Who Converted Prior to the Study Period [file jamanetwopen-e2342786-s002.pdf]

Supplemental Online Content

Gao E, Melnick ER, Paek H, Nath B, Taylor RA, Loza AJ. Adoption of emergency department–initiated buprenorphine for patients with opioid use disorder: a secondary analysis of a cluster randomized trial. *JAMA Netw Open*. 2023;6(11):e2342786. doi:10.1001/jamanetworkopen.2023.42786

eTable. Cox Proportional Hazards Model of Prescriber Conversion Based on Prior 4-Month Exposure Count[a]
eFigure 1. Study Flow Diagram
eFigure 2. Conversion of Others Exposed by an Index Clinician
eFigure 3. Rates of Subsequent Prescribing or Ordering Events
eFigure 4. Association Between Conversion to Buprenorphine Prescriber Status and Number of Exposures to Buprenorphine Prescribing Within the Last Four Months
eFigure 5. Sensitivity Analysis: Assessing Bias Introduced by Clinicians Who Converted Prior to the Study Period

This supplemental material has been provided by the authors to give readers additional information about their work.

© 2023 Gao E et al. *JAMA Network Open.*

eTable. Cox Proportional Hazards Model of Prescriber Conversion Based on Prior 4-Month Exposure Count[a]

**Variable**	**HR**	**95% CI**	**p-value**
Clinician Role (Relative to APP[b])			
Attending	0.54	0.34-0.85	**0.008**
Resident	0.49	0.30-0.79	**0.004**
Intervention Site Status (Relative to Usual Care)	1.81	1.11-2.93	**0.02**
Academic Site Status (Relative to Non-academic)	2.77	1.70-4.51	**<0.001**
Healthcare System (Relative to System 1)			
System 2	3.50	1.56-7.85	**0.002**
System 3	10.0	3.32-30.3	**< 0.001**
System 4	4.65	2.37-9.12	**< 0.001**
System 5	8.55	4.34-16.8	**< 0.001**

© 2023 Gao E et al. *JAMA Network Open.*

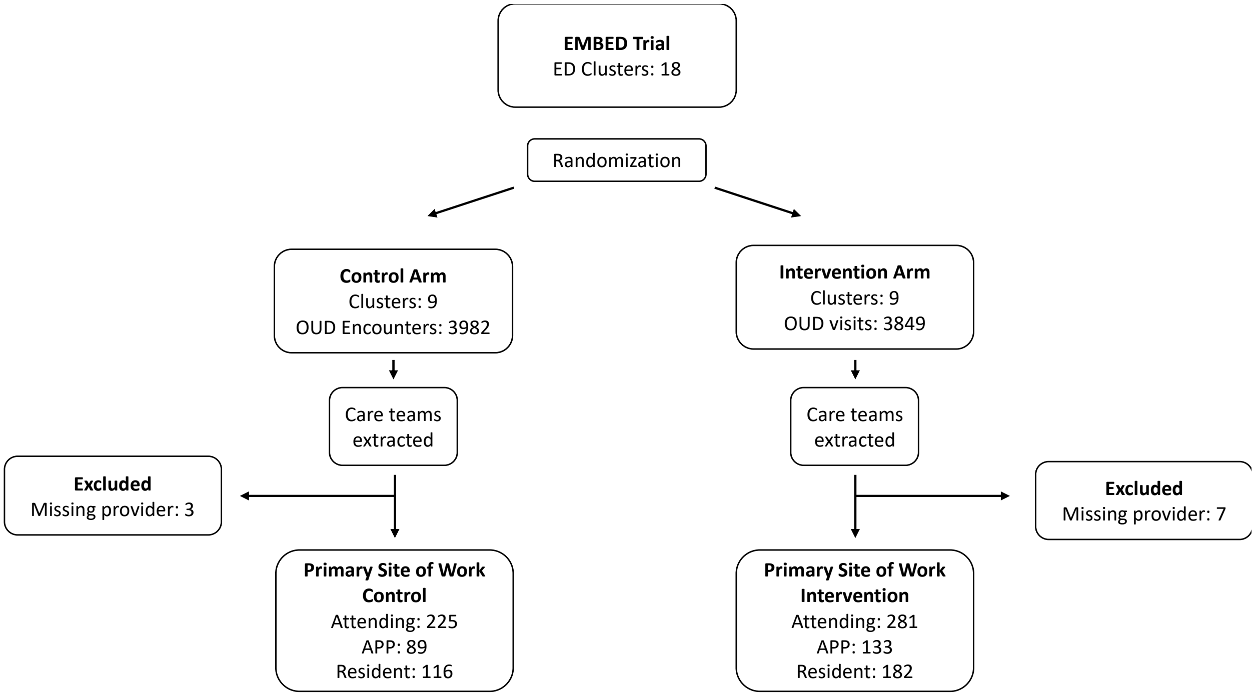

eFigure 1. Study Flow Diagram

© 2023 Gao E et al. *JAMA Network Open.*

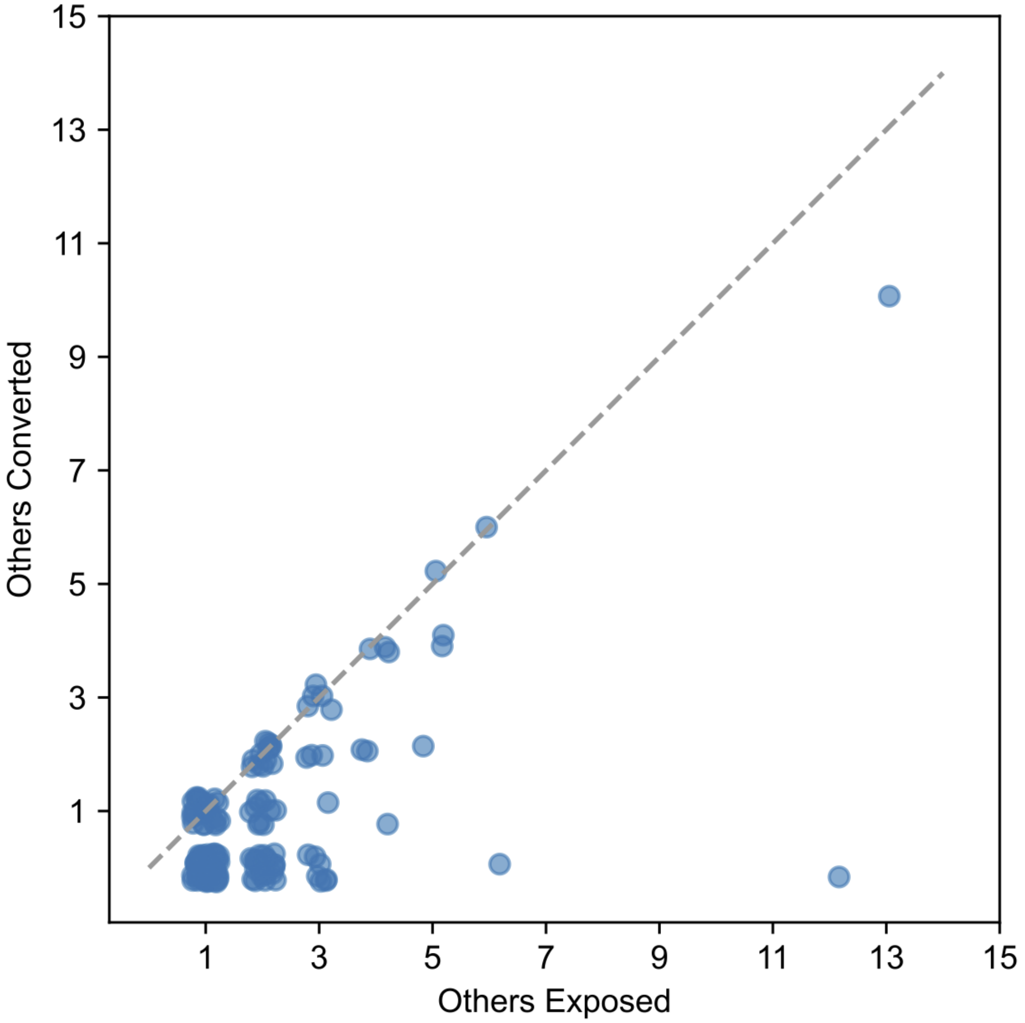

eFigure 2. Conversion of Others Exposed by an Index Clinician. The number of other clinicians exposed by an index clinician, before conversion of the other clinician, is shown on the x-axis. The number of these other clinicians who eventually converted is shown on the y-axis. The dashed gray line represents a 100% conversion rate where all others exposed by an index clinician end up converting within the study. Points have been jittered in the x and y direction due to the high degree of overlap.

© 2023 Gao E et al. *JAMA Network Open.*

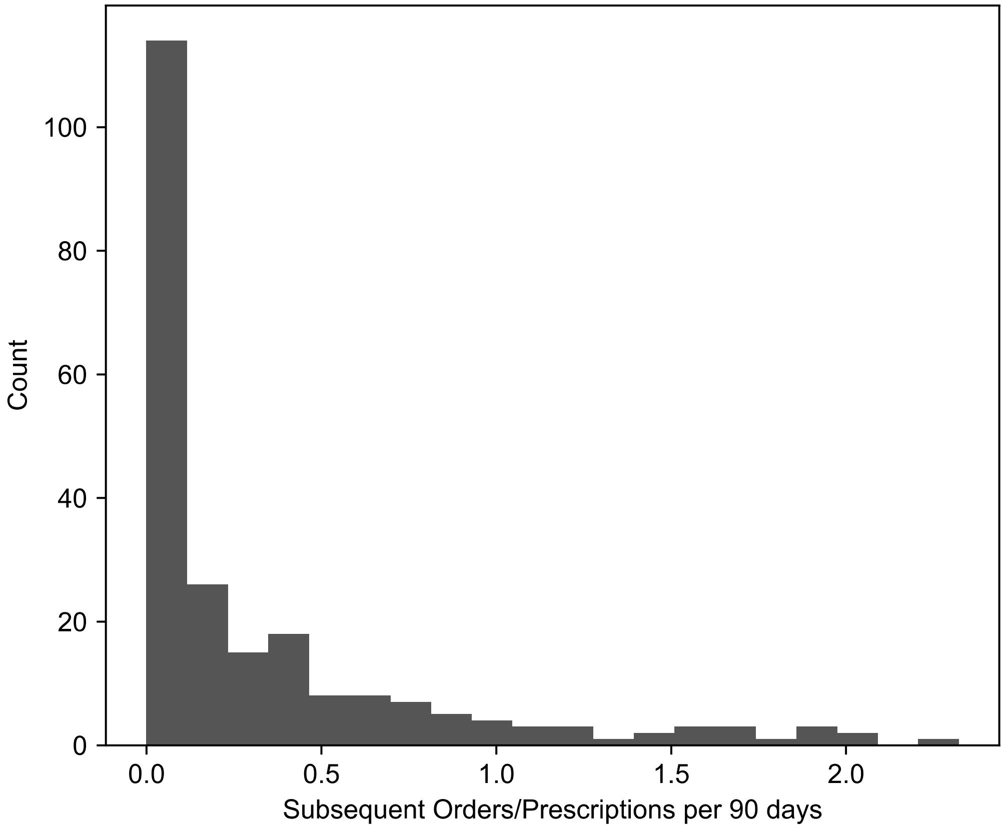

eFigure 3. Rates of Subsequent Prescribing or Ordering Events. For clinicians who converted, a histogram of the number of subsequent prescribing or ordering events divided by the remaining study time after conversion and then normalized to a 90-day period is shown. There are 49.8% of clinicians with any subsequent prescribing event.

© 2023 Gao E et al. *JAMA Network Open.*

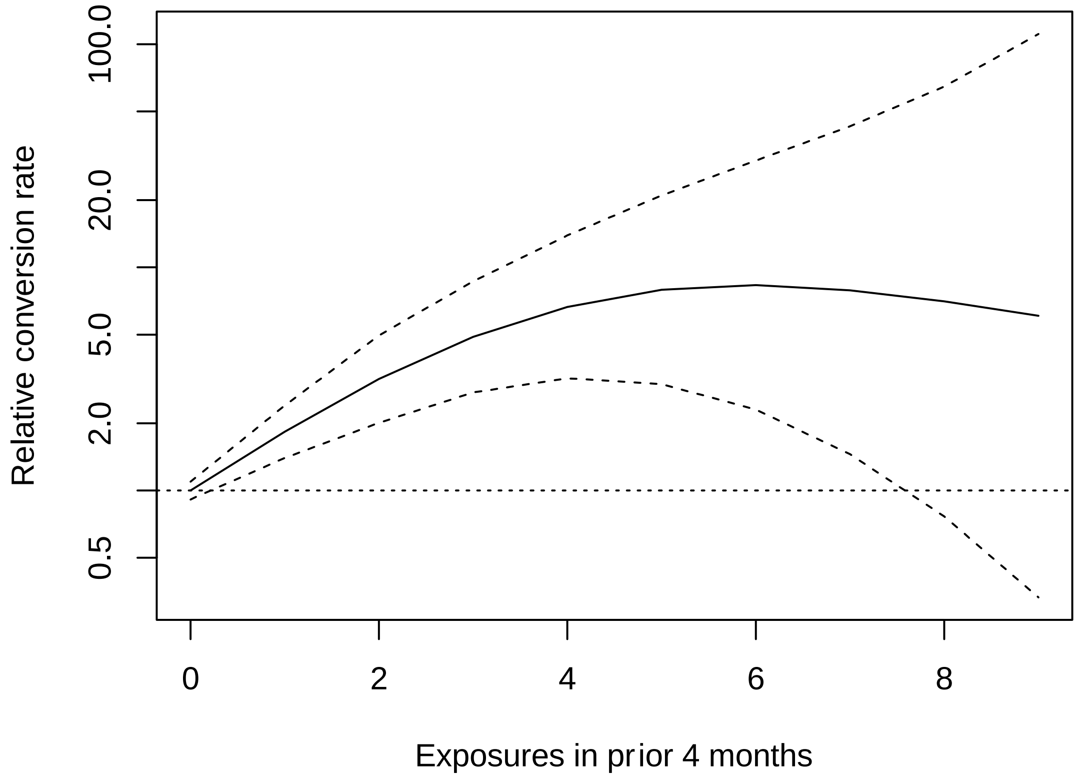

eFigure 4. Association Between Conversion to Buprenorphine Prescriber Status and Number of Exposures to Buprenorphine Prescribing Within the Last Four Months. Note that a log scale is used for tick spacing on the y-axis. The solid line shows the estimated relative conversion rate with 95%-CI envelope contained within the dashed lines.

© 2023 Gao E et al. *JAMA Network Open.*

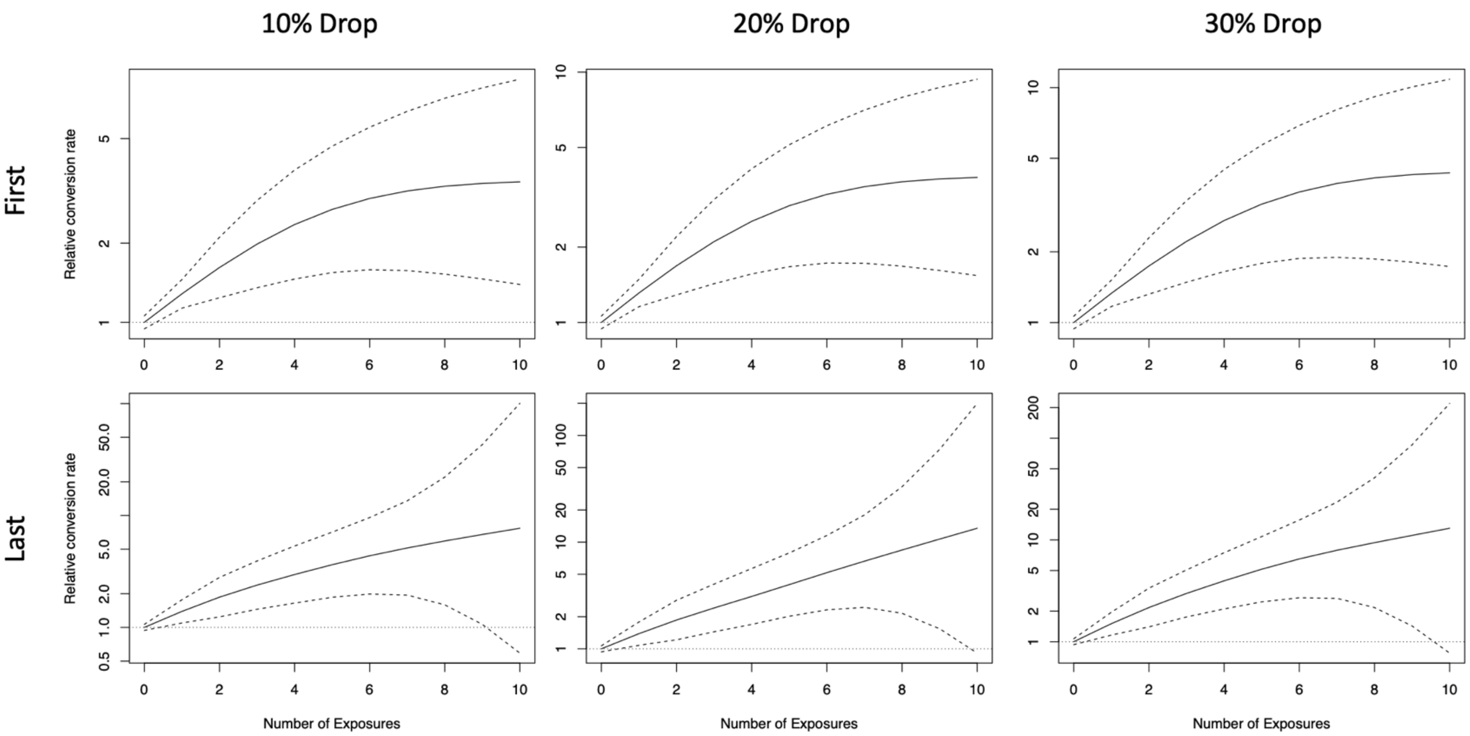

eFigure 5. Sensitivity Analysis: Assessing Bias Introduced by Clinicians Who Converted Prior to the Study Period. To assess for bias arising from including the small number of clinicians who may have converted prior to the study period, fractions of clinicians were removed from having the outcome but were still able to expose others. Testing two hypotheses, the first and last 10, 20, and 30% of clinicians were removed. The dose dependent hazard rate of conversion as a function of exposure count is shown.

© 2023 Gao E et al. *JAMA Network Open.*
