# Supplementary material for: Adoption of Emergency Department–Initiated Buprenorphine for Patients With Opioid Use Disorder: Secondary Analysis of a Cluster Randomized Trial
Source: JAMA Netw Open. 2023 Nov 10;6(11):e2342786. doi: 10.1001/jamanetworkopen.2023.42786 (PMC10638655; doi:10.1001/jamanetworkopen.2023.42786)
Supplement: Supplement 3. — Data Sharing Statement [file jamanetwopen-e2342786-s003.pdf]

Data Sharing Statement

Gao. Adoption of Emergency Department–Initiated Buprenorphine for Patients With Opioid Use Disorder. *JAMA Netw Open*. Published November 14, 2023.
doi:10.1001/jamanetworkopen.2023.42786

Data

Data available: Yes
Data types: Deidentified participant data, Data dictionary
How to access data: Data Available: yes Type: data deidentified at patient, provider, and site level; data dictionary How to access: - EMBED trial Public repository: https://www.icpsr.umich.edu/web/NAHDAP/studies/38568/summary - Care team data available via andrew.loza@yale.edu When available: since 11/2022
When available: With publication

Supporting Documents

Document types: Statistical/analytic code
How to access documents: Supporting documents Available: yes Types: statistical/analytic code How to access: andrew.loza@yale.edu When available: beginning 6/1/2023
When available: With publication

Additional Information

Who can access the data: Who can access the data: public dataset is available. For additional data on care teams, researchers whose proposed use of the data has been approved by the EMBED team
Types of analyses: For a specified scientific purpose
Mechanisms of data availability: After approval of a proposal
